# Elucidation of collagen content in different anatomical regions of the dermis of donkeys (*Equus asinus*): histomorphometric and ultrastructural study

**DOI:** 10.1186/s12917-025-04712-0

**Published:** 2025-05-02

**Authors:** Yasmeen Magdy, Ahmed Abo-Ahmed, Mohamed Abumandour, Anwar El Shafey, Reda El-Kammar, Mai A. AL-Mosaibih, Eman Fayad, Osama Ahmed

**Affiliations:** 1 Department of Anatomy and Embryology, Faculty of Veterinary Medicine, Benha University, Toukh, 13736 Egypt; 2 Department of Anatomy and Embryology, Faculty of Veterinary Medicine, Alexandria University, Post Box: 22758, Abees 10th, Alexandria, 21944 Egypt; 3 Department of Histology, Faculty of Veterinary Medicine, Benha University, Toukh, 13736 Egypt; 4 University of Jeddah, College of Science, Department of Biological Sciences, Jeddah, Saudi Arabia; 5 Department of Biotechnology, College of Sciences, Taif University, P.O. Box 11099, Taif, 21944 Saudi Arabia

**Keywords:** Donkey, Skin, Collagen, TEM, Dermo-epidermal interface, Accessory cordovan layer

## Abstract

Research on collagen content in donkey skin is limited, necessitating experimental approaches to aid in the economic production of the leather and cosmetics industries. This study aimed to analyze the histomorphometry and ultrastructure of donkey skin and compare collagen fiber zones in various anatomical regions, both objectively and quantitatively. The study was the first to describe the melanosome number in different skin regions of a donkey. Skin biopsies from fourteen mature male donkeys were used in this study. The epidermis and dermis were examined histologically and ultrastructurally, focusing on the dermo-epidermal thickness ratio and collagen bundle arrangement in the dermis. The study revealed that melanin deposits are higher in the limbs and abdomen of donkeys compared to the back, neck, and thorax, and the dermal-epidermal junction of the back skin is longer. The back skin is the thickest, while the limb skin is the thinnest, despite the high collagen content in the thoracic region. The study indicates that the variation in melanin deposits and dermal-epidermal junction lengths in different body regions of donkeys may be due to variations in mechanical stress and environmental factors. The abdomen had the highest melanocyte count, with significant differences compared to the neck, back, and thorax. The neck region had the lowest count, while the limb, back, and thorax had similar counts. The study’s findings could aid in identifying the difference between normal skin features and collagen-diseased skin in donkeys, aiding in diagnosis and providing valuable insights for veterinarians and researchers studying similar conditions.

**Supplementary Information:**

The online version contains supplementary material available at 10.1186/s12917-025-04712-0.

## Highlights

Our study aims to understand the histological composition and collagen content of donkey skin in various anatomical regions like the chest, abdomen, back, and limbs. The study aims to identify areas of donkey skin suitable for use in the leather and cosmetics industries. Different areas of donkey skin were studied using light and electron microscopy, and we statistically found that the highest amount of collagen is found in the skin of the chest. The histomorphometric features of the donkey’s skin offer additional dermatological evaluation and valuable tools for assessing skin lesions.

**Supplementary Information:**

The online version contains supplementary material available at 10.1186/s12917-025-04712-0.

## Introduction

Donkeys, domesticated members of the *Equidae*, were used as working animals, particularly in underdeveloped countries, for draft and riding purposes in rural areas. The largest number of donkeys lives in developing countries. An anticipated 59 million donkeys as well as mules exist worldwide [1]. The donkey, a common domestic livestock animal, was used for farming and providing sustenance in certain communities due to its economical transportation status [2, 3]. In 2014, Egypt had 3.2 million donkey heads, but now it’s only one million. This decline is attributed to the Chinese market’s demand for donkey hides, overexploitation of hides, and industrialization, as well as the growing demand for Egyptian donkeys for preparation and industrialization [4–6].

The skin, constituting about 16% of the body mass, serves as the first barrier between the body and its environment, protecting and maintaining homeostasis [7–9]. Szczepanik, et al. [10] asserted that the strong skin tear resistance during technological processing is facilitated by the proper dermal and epidermal structure, as well as subcutaneous tissue. Skin histomorphometry measures the thickness of skin layers, collagen and elastic fiber bundles, and skin accessories like sweat glands, sebaceous glands, and hair follicles. This quantitative evaluation provides specific physiological functions for donkeys to adapt to environmental changes [11] and defines the quality of their skin [12]. *Colla corii asini* (E′jiao, A’jiao) was created using the skin of *Equus asinus L.* as the primary raw material. A gelatin-like block-shaped preparation called *Colla corii asini* yielded 58 compounds, mostly proteins, amino acids, polysaccharides, gelatins, inorganic substances, volatile substances, and so on [13].

Collagen is a fibrous, insoluble protein present in the skin and other tissues of the body in all species. In industry, collagen has been widely used for the production of gelatin, especially in the pharmaceutical and cosmetic industries [14]. The texture of skin is ensured by collagen, and as we age, our bodies produce less collagen annually by 1–2%, starting at age thirty [15]. Also, different collagen phenotypic characteristics of body and limb skin can reflect dissimilarities in healing patterns [16], so there is a persistent need for collagen evaluation and quantification [17].

There is currently very little scientific information about donkey skin in research, emphasizing the need to create a fundamental structure, determine collagen distribution, and establish quantifiable information that might be valuable to E′jiao manufacture. Additionally, this study describes the histomorphometry aspects of a donkey’s skin that provide additional dermatological evaluation and useful tools for assessing the skin lesions. Understanding the histomorphometry aspects of a donkey’s skin can contribute to a better elucidation of its structure and potential applications in the field of dermatology. Furthermore, this research can provide valuable insights into the development of treatments for skin lesions in donkeys and potentially other animals with similar skin characteristics.

## Material & methods

### Animals and samples

This study was carried out at the Anatomy and Embryology Department, College of Veterinary Medicine, Benha University, Egypt. The Institutional Animal Care and Use Committee of Benha University and the National Institute of Health (NIH) guidelines in Egypt (**Ethical No. BUFVTM 12-02-22**) accepted all procedures used in this study. Skin samples from 14 adult donkeys (125–135 kg), client-owned and euthanized for unrelated reasons, were collected for this study. The examined skin samples were collected from donkeys during fall and winter 2020 with no history of skin injuries, disorders, or abnormalities. During mid-morning, the skin samples were immediately obtained once each donkey had been euthanized. The skin locations were clipped and treated with chlorhexidine digluconate, 2% germicide, and a 0.5% alcoholic solution. Each skin specimen underwent a thorough dissection to expose the subcutaneous fat layer beneath. Skin samples’ size was 2 × 2 cm for histological examination and 0.5 × 0.5 cm for electron microscopy (TEM). Skin specimens were taken from five various parts of the donkey’s cadaver (neck, thorax, back, caudal abdomen, and limb).

### Light microscopic studies

The skin tissue samples (0.5 cm3) were immediately well-kept in 10% formalin (neutral buffered) to be fixed and prepared for the different histological techniques [18]. After 24 h, the samples were extensively transferred to 70% alcohol. The tissue samples were then dehydrated in an ascending grade of ethanol, cleared in xylene, and impregnated and embedded in paraffin wax. Sections of 5 μm were cut using a Leica rotatory microtome (*RM 20352035; Leica Microsystems*,* Wetzlar*,* Germany*) and mounted on glass slides. Paraffin sections were prepared according to the protocol of hematoxylin and eosin (H&E) staining [18, 19] for general histology. For collagen and muscle fiber staining, Masson’s trichrome [20, 21] was used. The sections were examined and photographed under a bright field light microscope (*Olympus BX 50 compound microscope*).

### Transmission electron microscopy

The skin was prepared for TEM examinations, according to Kandyel, et al. [22]. Skin specimens were fixed for four days at 4 °C in a combination of 2.5% paraformaldehyde - glutaraldehyde solutions. The specimens (each about 1 mm^3^) were first fixed by submersion in a solution containing 5% glutaraldehyde and 0.1 M sodium cacodylate buffer. Subsequently, they were fixed by rinsing in a solution containing 1% osmium tetroxide and 0.1 M sodium cacodylate buffer. The samples underwent 15 min of dehydration in escalating ethanol grades of 60, 70, 80, 90, and 100%. The dehydrated samples were then soaked in epoxy resin for one hour at 40 °C. For light microscopy, the semithin sections with 1 μm thickness were prepared and stained with 1% toluidine blue. Next, for electron microscopy, 60 nm ultra-thin sections were made and stained with uranyl acetate, followed by lead citrate. The stained ultra-thin sections were investigated by using a transmission electron microscope (JEOL 1010) at the electron microscopy unit, Faculty of Science, Alexandria University, Egypt.

### Morphometric measurements

Quantitative measurements were done using Leica’s morphometric software suite version 4.0 (LAS V4.0). For every animal, 5 random sections stained with H&E were investigated with a 10× objective lens. Quantitative analysis included measurements of both epidermal and dermal thickness as well as the distance of the dermo-epidermal junction. Using computer software, a line was drawn along the dermal epidermal interface to determine its extent. In addition to those measurements, the quantity of sebaceous glands, hair follicles, sweat glands, arrector pili muscles, and collagen contents in the dermis was calculated for each slide, and the dermis area was measured, and these data were presented as the mean number per mm^2^. Images were captured at five different magnifications: four, ten, twenty, forty, and one hundred. At all magnifications, qualitative elucidation of tissue architecture and cell morphology was carried out. All measurements were done using ImageJ software [23].

### Statistical analysis

To display the results, the means and standard errors of the mean (TEM) were calculated. The results were calculated using a T-test, ordinary one-way analysis of variance (ANOVA) for normally distributed data, and ANOVA on rank for non-normally distributed data at P 0.05, the acquired data were deemed statistically significant. The data was analyzed with *GraphPad Prism 9 software*, and the results were displayed as means ± histology. The mean thickness (µm) of epidermis and dermis; length of dermo-epidermal interface (µm), and Number of hair follicle muscles/mm^2,^ sebaceous gland clusters/mm2, sweat gland clusters/mm2, and arrector pili muscles/mm2; Content of collagen.

## Results

### Light microscopic findings

#### Epidermis

**Fig. 1:**
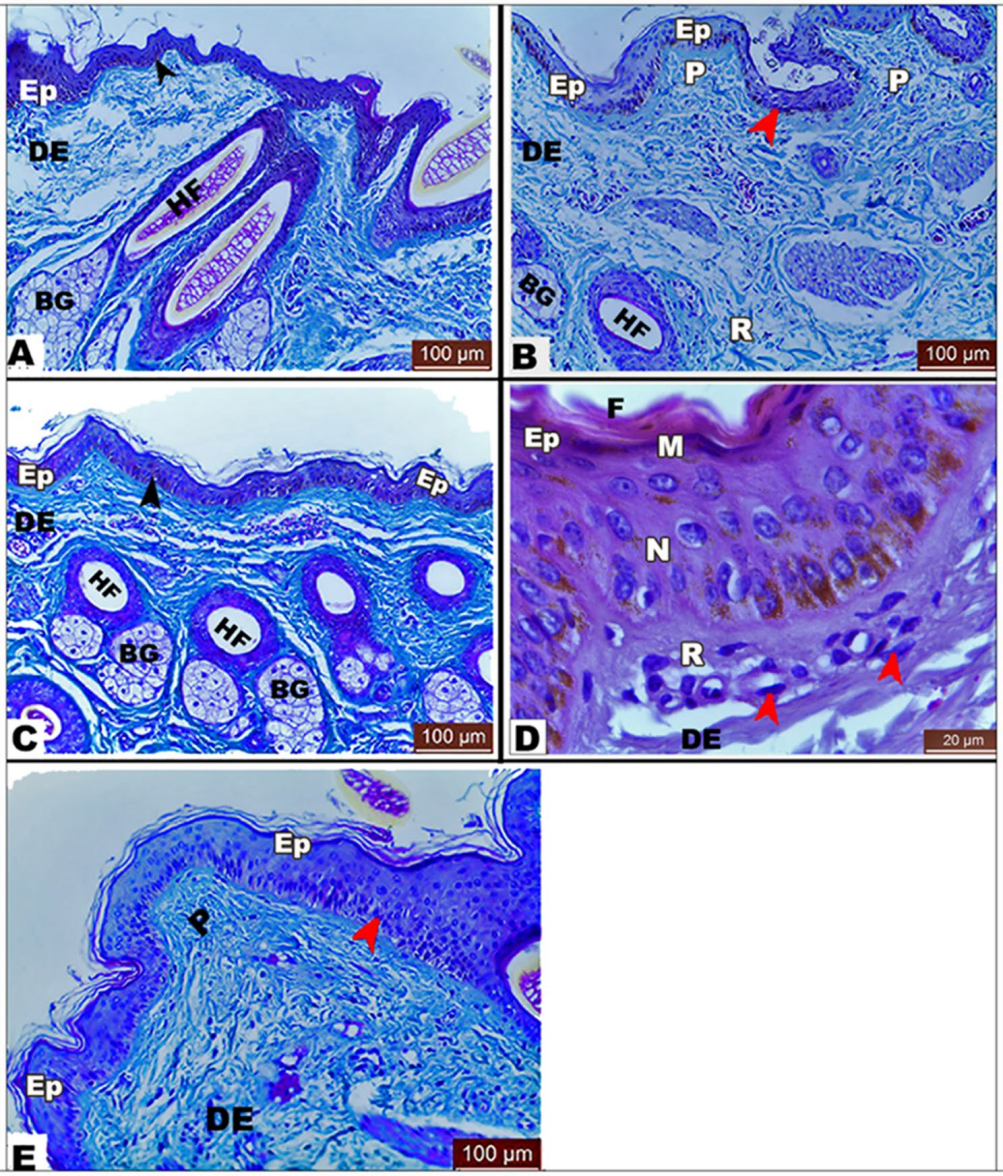
Photomicrograph of *Equus asinus* skin showing normal histological structure of the abdominal region with serrated dermo-epidermal junction (black arrowhead) in (View **A**), the back region with melanocytes with a low number (red arrowhead) in (View **B**), the limb region with its flattened dermo-epidermal junction (black arrowhead) in (View **C**), the neck region with melanocytes and a low number (red arrowhead) in (View **D**), and the thorax region with melanocytes with a low number (red arrowhead) in (View **E**). *Abbreviations*: epidermis (Ep), dermis (DE), papillary dermis (P), reticular dermis (R), stratum corneum (**F**), stratum granulosum (**M**), stratum spinosum (**N**), and stratum basale (R). *Masson trichrome stain; bar indicates magnification*

There were four layers in the thin epidermis of every anatomical region, including the neck, back, thorax, abdomen, and limb, namely the stratum basale, stratum spinosum, stratum granulosum, and stratum corneum. The stratum basale of the epidermis was characterized by cuboidal or low columnar cells with ovoid or round nuclei resting on a wavy basement membrane. It was also observed that there were several layers of large polyhedral cells in the stratum spinosum. Moreover, the stratum granulosum contained two to three layers of flattened squamous cells. In addition, it was found that the outer layer was the stratum corneum (horny layer), as described in (Fig. 1). Melanocytes were observed in low numbers within the stratum basale of the back, neck, and thorax regions and in high numbers in the abdomen and limb regions (Fig. 1).

**Fig. 2:**
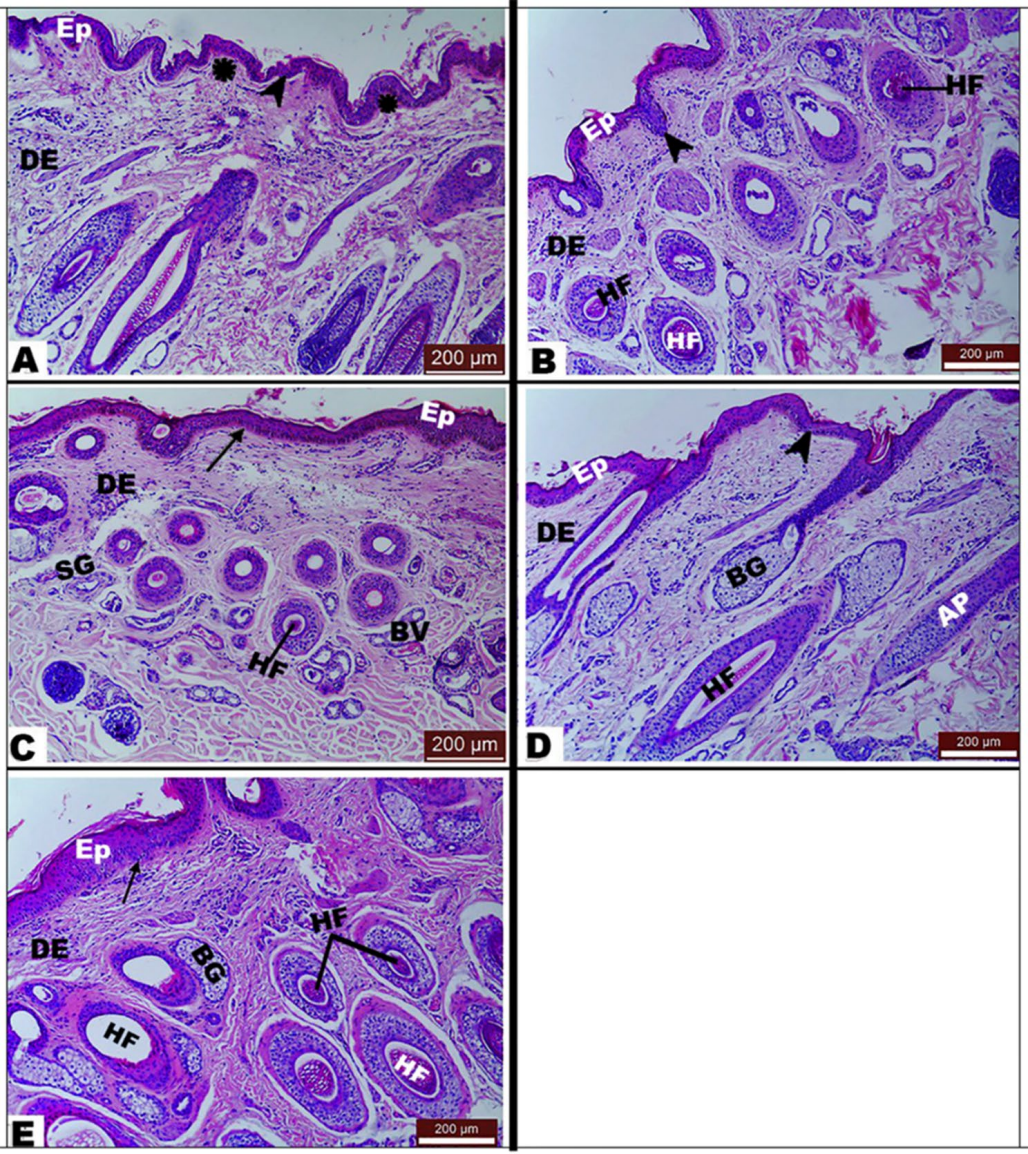
Photomicrograph of *Equus asinus* skin showing normal histological structure of the abdominal region (View **A**), the back region (View **B**), the limb region (View **C**), the neck region (View **D**), and the thoracic region (View **E**). *Abbreviations*: epidermis (Ep), dermis (DE), hair follicle (HF), sweat gland (SG), sebaceous gland (BG), *a* black arrow indicated a dermo-epidermal junction, a black arrowhead indicated epidermal pillars, and a black star indicated dermal papillae. *H&E stain; bar indicates magnification*

Basal cells in the epidermal part atop the apices of the dermal papillae gave rise to various villous cytoplasmic protrusions in the dermis, giving rise to a serrated appearance in the abdomen, back, and neck regions. These prominent, tiny foot-like protrusions or processes created a greatly tortuous dermo-epidermal junction. However, the basal cells of the skin of the limbs lost these protrusions, giving a flattened dermo-epidermal junction (Fig. 2).

The epidermal part at the top apices of the dermal papillae gave rise to various villous protrusions in the dermis, giving rise to a serrated appearance in the abdomen, back, and neck regions. These prominent, tiny foot-like protrusions or processes created a greatly tortuous dermo-epidermal junction. However, the epidermis of the skin of the limbs lost these protrusions, giving a flattened dermo-epidermal junction (Fig. 2).

#### Dermo-epidermal interface

It constituted support for epidermal cells; in all anatomical regions, the dermo-epidermal junction was wavy, with well-defined columns of dermis expanding in-between dermal papillae (Fig. 2).

#### Dermis

In all the areas of the skin, there were two layers of dermis: the outer narrow papillary vascular dermis and the wider inner reticular dermis. The use of Masson trichrome to stain the dermis allowed clear identification of a collagenous network. On the other hand, collagen fibers were of comparable size and distribution in all samples (Fig. 1).

**Fig. 3:**
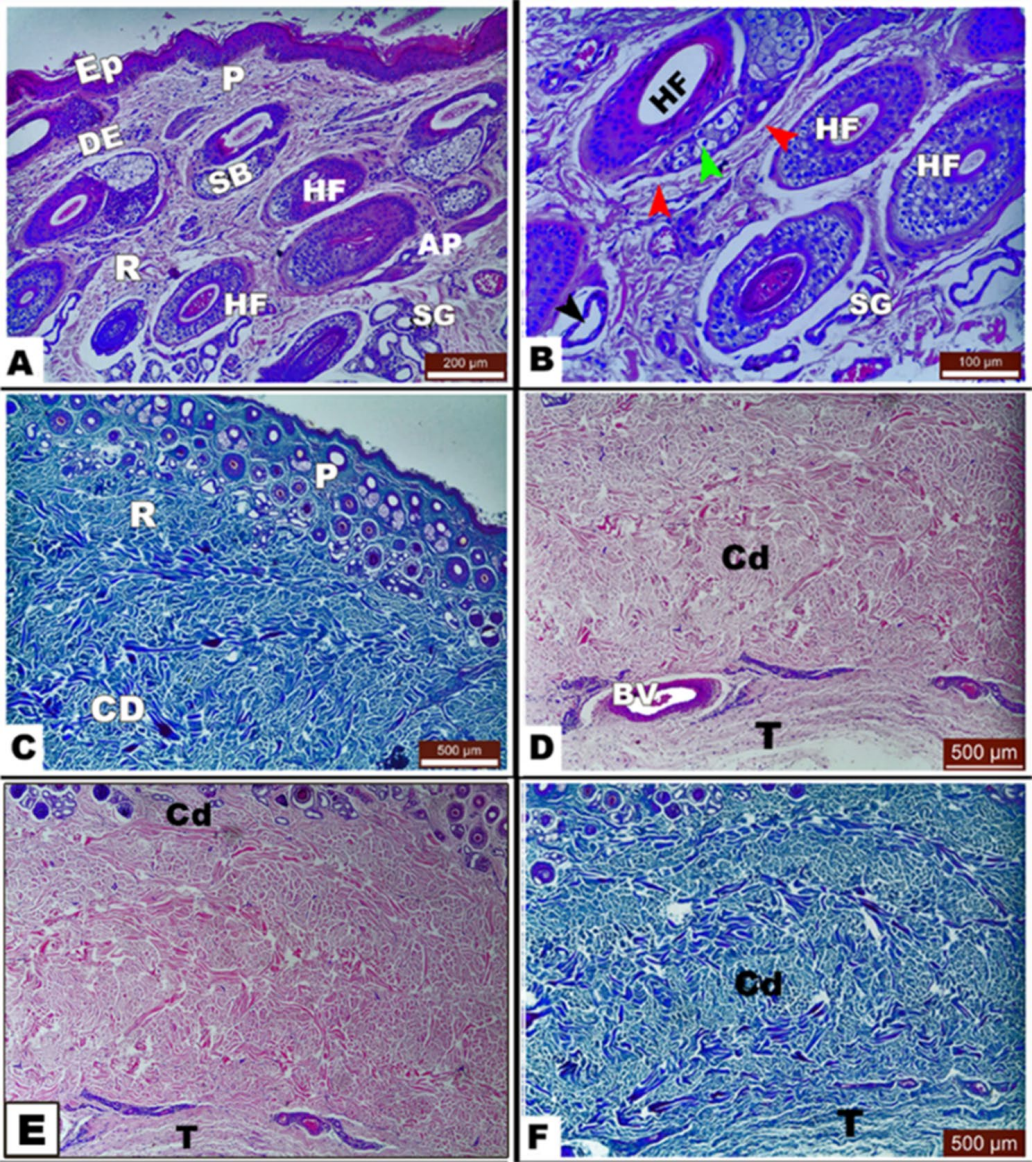
Photomicrograph of *Equus asinus* skin. (View **A**) showing skin appendages in different anatomical regions with *H&E stain*, (View **B**) with *H&E stain*, (View **C**) with *Masson trichrome stain*, (Views E and F) showing the orientation of collagen fibers in the third accessory layer (T) parallel to the epidermis cordovan layer (Cd) with *H&E stain* in (View E), and *Masson trichrome stain* in (View F). ***Abbreviations***: epidermis (Ep), dermis (DE), arrector pilli muscle (AP), hair follicle (HF), sebaceous gland (SB), sweat gland (SG), papillary dermis (P), reticular dermis (R), hair follicle (HF), myoepithelial cells (red arrowhead), sebaceous gland cells (green arrowhead), multivacuolated cytoplasm (black arrowhead) with central nucleus, and blood vessels (Blvs)

The reticular layer of the dermis is home to skin appendages like sebaceous glands, hair follicles, sweat glands, and arrector pili muscles. In various anatomical regions, sebaceous glands with sacculations accompanied the oval-shaped hair follicles. Sebaceous glands were simple, branched, and showed a multi-bubble cytoplasm with central nuclei. Sweat glands were found in the core of the reticular dermis and were coiled tubular with a secretory low-cuboidal epithelium with acidophilic cytoplasm and round basally located nuclei. The arrector pili muscle began as a small bundle of smooth muscle near the epidermis and was later divided into as many branches at the sebaceous gland (Fig. 3A and C).

It was observed that the dermis contains thin and thick collagen bundles in the papillary dermis and reticular dermis, respectively. The cordovan layer was detected under the reticular dermis, while collagen bundles appeared thicker and had an accessory layer (third layer) in the limbs only (Fig. 3). The accessory layer’s collagen fibers were aligned parallel to the epidermis.

### Transmission electron microscopy

#### Epidermis

**Fig. 4:**
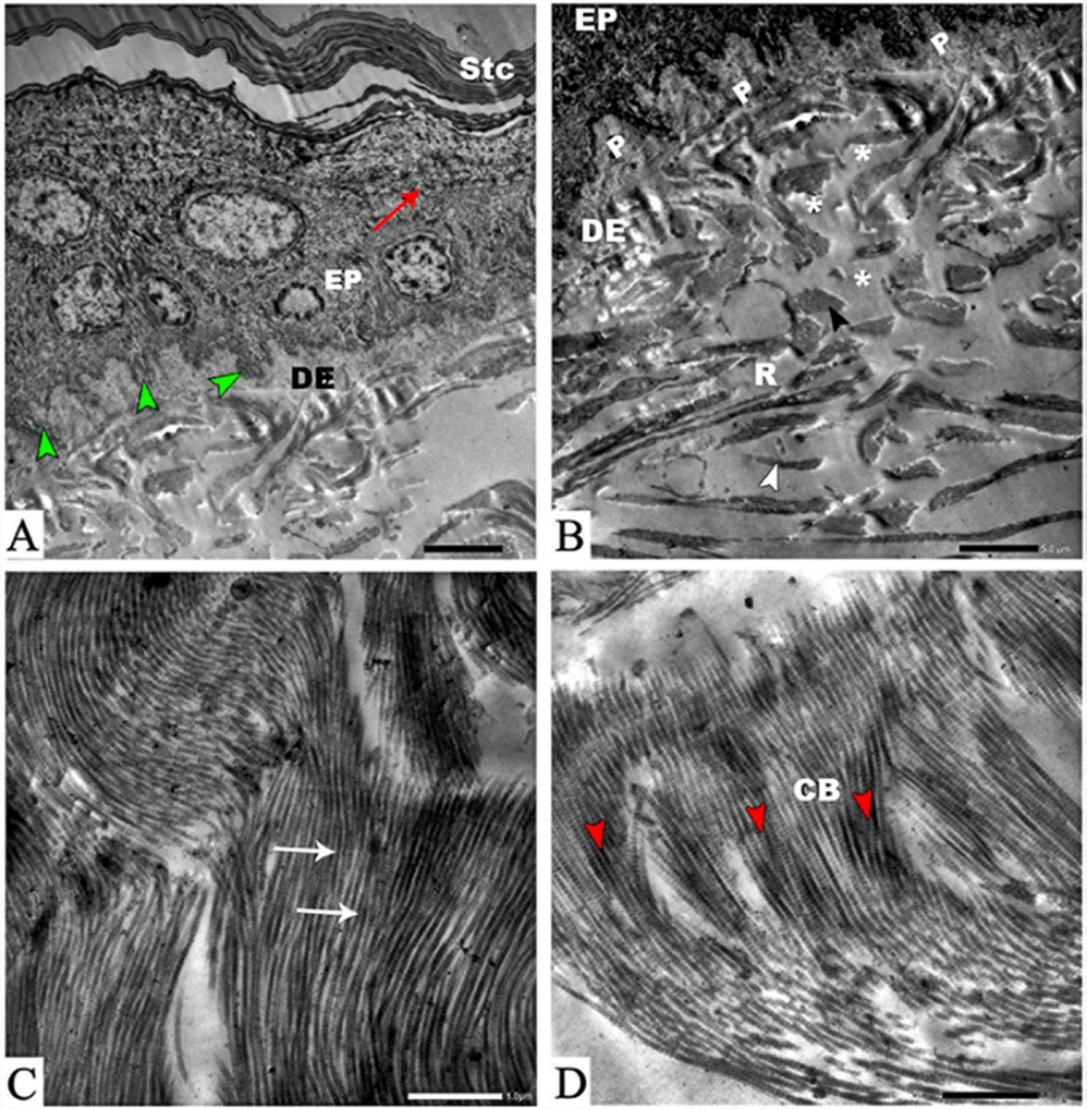
Transmission electron micrograph of *Equus asinus* skin. (View **A**) shows the structure of the epidermis with wavy stratum corneum (Stc), granules of keratohyalin (red arrows), epidermis (EP), dermis (DE), and finger-like projections of basal cells (green arrowheads). (View **B**) shows the organization of collagen in the dermis (DE): papillary dermis (P), the epidermis (EP), reticular dermis (R), and dermal collagen sectioned transversely and longitudinally (white arrowheads); white stars indicate electron lucent spaces. (View **C**) shows the arrangement of collagen in the dermis (white arrows). (View **D**) showing the bundles of fine elastin microfibrils (red arrowheads) that were inserted between collagen bundles (CB)

The stratum corneum presented a wavy appearance upon ultrastructural observation of the limb’s epidermis. The fundamental characteristics of the stratum granulosum include the presence of intracellular keratohyalin granules. Basal cells were distinguished by their cell membranes containing finger-like protrusions that extend into the dermis and form finger-like protrusions of basal cells. Moreover, the wavy dermo-epidermal junction was noticed (Fig. 4A).

#### Dermis

Collagen was arranged into dense masses oriented mainly parallel to the surface of the skin. Dermal collagen appears transversely and longitudinally cut into the dermis. The papillary dermis consisted of a “lacy-like” arrangement of fibers with prominent electron spaces between the fibrils, as the collagen appeared as a cluster of randomly distributed microfibrils and small bundles in the papillary dermis (Fig. 4B). Large undulating, loosely interwoven collagen bundles were found in the reticular dermis; these bundles were randomly oriented (Fig. 4C). On the other hand, the fibers within a particular bundle were densely packed together. Microfibrils of elastin were seen inserted between the collagen bundles (Fig. 4D).

### Morphometric and statistical analysis of the measurements of skin

**Fig. 5:**
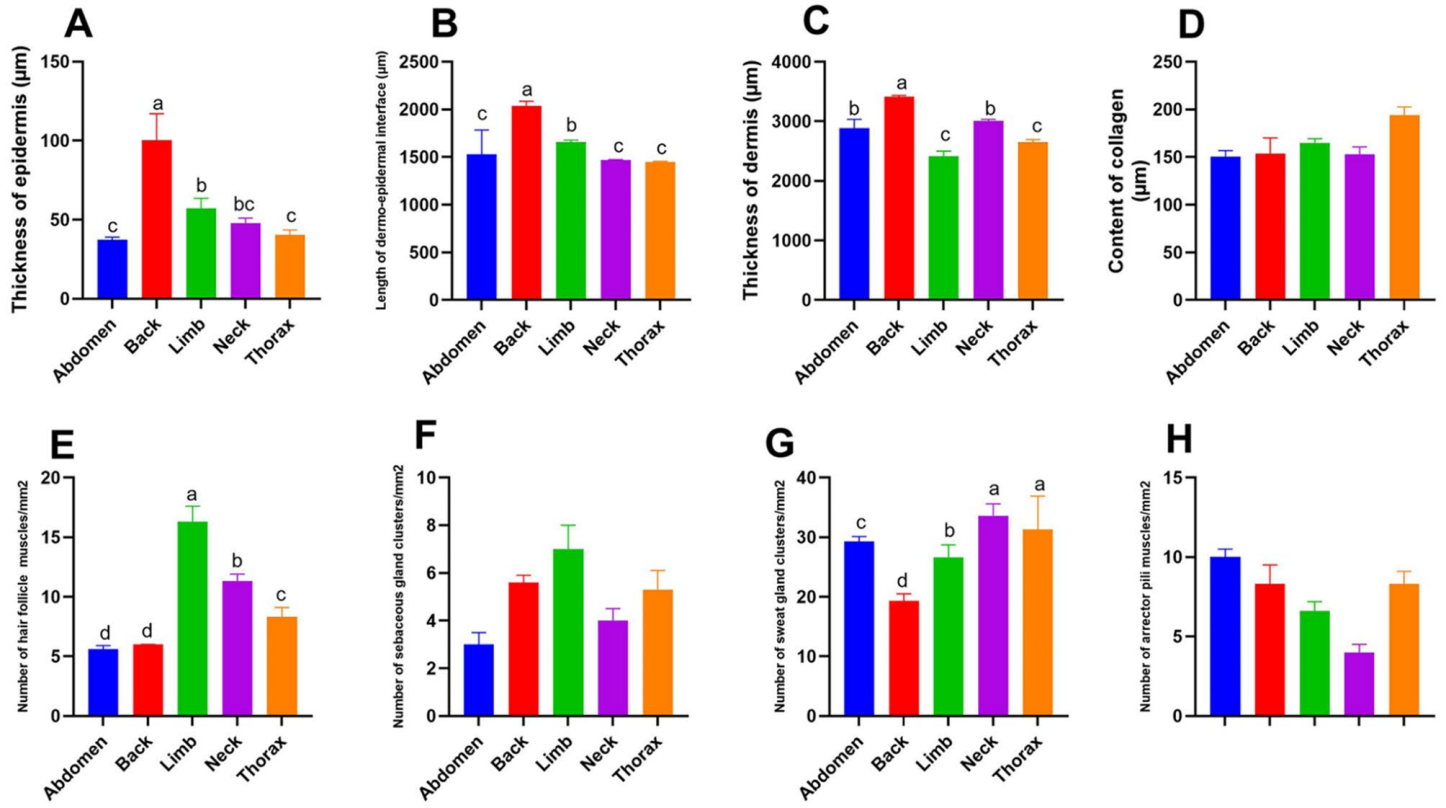
Graph describing the measurements of different parameters (mean ± SE) of donkey’s skin, including the epidermis thickness (View **A**), the dermo-epidermal interface length (View **B**), dermal thickness (View **C**), the collagen content of the dermis (View **D**), the hair follicles’ number in the dermis (View **E**), the sebaceous and sweat glands’ number in the skin (View **F**-**G**), and the number of arrector pili muscles (View **H**)

The epidermis thickness ranged from 37.3 to 100.2 μm. In the neck, thorax, and abdomen, the epidermal thickness ranged from 37.3 to 47.9 μm. While in the back and limb, the epidermis was relatively thick (100.2 ± 16.8 μm), as described in Table 1 and (Fig. 5A). It was found that the thickness of the skin in all five areas, which include the neck, back, chest, abdomen, and limbs, differs significantly (*p* = 0.003), as described in (Table 1) and (Fig. 5A).

**Table 1:** Measurements of different parameters (mean ± SE) of Donkey’s skin, including **t**he epidermis thickness, the dermo-epidermal interface length, and dermal thickness, the collagen content of the dermis, the hair follicles’ number in the dermis, the sebaceous and sweat glands’ number in the skin, and the number of arrector pili muscles

Parameter	Abdomen *N* = 14	Back *N* = 14	Limb *N* = 14	Neck *N* = 14	Thorax *N* = 14	*P* value
Thickness of epidermis (µm)	37.3 ± 1.7	100.2 ± 16.8	57.3 ± 6.3	47.9 ± 3.1	40.0 ± 3.3	0.003
Length of dermo-epidermal interface (µm)	1531 ± 253.9	2037.3 ± 50.2	1656.9 ± 20.3	1470.4 ± 4.7	1450.7 ± 6.2	0.021
Thickness of dermis (µm)	2883.4 ± 150.6	3414.8 ± 20.1	2415.5 ± 85.1	3005.1 ± 30.9	2653.2 ± 40.7	0.011
Content of collagen (µm)	150.3 ± 6.5	153.6 ± 16.5	164.9 ± 4.4	153.3 ± 7.5	194.2 ± 8.3	0.205
Number of hair follicle muscles/mm^2^	5.6 ± 0.3	6 ± 0.01	16.3 ± 1.3	11.3 ± 0.6	8.3 ± 0.8	0.014
Number of sebaceous gland clusters/mm^2^	3 ± 0.5	5.6 ± 0.3	7 ± 1	4 ± 0.5	5.3 ± 0.8	0.328
Number of sweat gland clusters/mm^2^	29.3 ± 0.8	19.3 ± 1.2	26.6 ± 3.5	33.6 ± 2.0	31.3 ± 5.6	0.027
Number of arrector pili muscles/mm^2^	10 ± 0.5	8.3 ± 1.2	6.6 ± 0.6	4 ± 0.5	8.3 ± 0.8	0.435

The dermo-epidermal interface length varied from 1450.7 to 2037.3 μm. The length in the abdomen, neck, and thorax ranged from 1450.7 to 1531 μm. The back and limbs were relatively long (1656.9–2037.3 μm), as seen in (Table 1) and (Fig. 5B). The length of the dermo-epidermal junction varied significantly between the donkey’s various anatomical regions (*p* = 0.021).

The dermal thickness ranged from 2415.5 to 3414.8 μm. In the abdomen, thorax, and limb, the thickness ranged from 2415.5 to 2883.4 μm, while the back and neck were relatively thick (3005.1–3414.8 μm). It was noticed that the back was the thickest (3414.8 μm), as described in Table 1 and (Fig. 5C). The anatomical regions of the donkey differed significantly in terms of skin thickness (*p* = 0.011).

The collagen content of the dermis varies from 150.3 to 194.2 μm, and that in the abdomen, back, and neck ranged from 150.3 to 153.6 μm. The thorax and limb were relatively thick (164.9–194.2 μm), as shown in (Table 1) and (Fig. 5D). No great difference was noticed between the dermal content of collagen between the different anatomical regions (*p* = 0.2).

The hair follicles’ number in the dermis varied from 5.6 to 16.3. The quantity of hair follicles varied significantly amongst the donkey’s five regions (*p* = 0.014), as shown in (Table 1) and (Fig. 5E). The sebaceous glands’ number in the skin of the abdomen was lower than in other regions; however, the variations between the regions were not significant (*p* = 0.32) whereas there was a statistically significant difference in the quantity of sweat glands across the donkey’s five skin regions (*p* = 0.02), as shown in (Table 1) and (Fig. 5F-G). Regarding the number of Arrector pili muscles, it was 4 in the neck’s skin, while in the abdomen, it was 10, however, they were not statistically different between the five regions of the donkey (*p* = 0.43) as shown in (Table 1) and (Fig. 5H).

### Statistical analysis of melanocyte in different skin regions

**Fig. 6:**
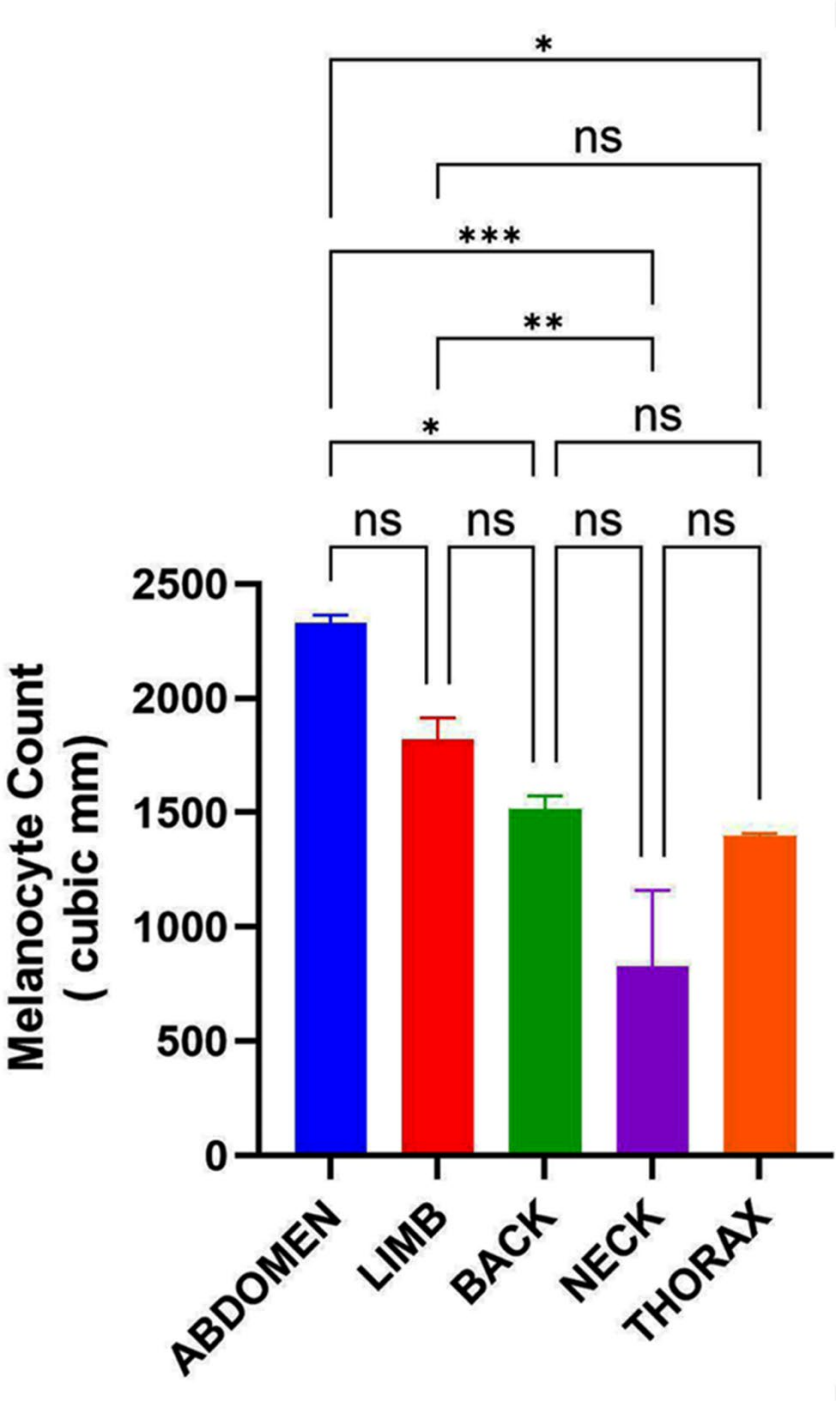
Graph describing the statistical analysis of melanocytes in different skin regions, including **t**he abdomen, limb, back, neck, and thorax. Abdomen is significantly higher than back, neck, and thorax (*). Abdomen is highly significantly higher than limb (*). The limb also shows a significant difference compared to the neck (**). (ns) is not significant; (*) is a significant difference at (typically *p* < 0.05). (*) and (**) are higher levels of significance (e.g., *p* < 0.01 and *p* < 0.001, respectively)

The statistical analysis of melanocytes in different skin regions, including the abdomen, limb, back, neck, and thorax. Abdomen is significantly higher than back, neck, and thorax (*). Abdomen was highly significantly higher than limb (*). The limb also showed a significant difference compared to the neck (**). (ns) is not significant; (*) was a significant difference at (typically *p* < 0.05). (*) and (**) are higher levels of significance (e.g., *p* < 0.01 and *p* < 0.001, respectively), as described in (Fig. 6).

Melanocytes were observed in low numbers within the stratum basale of the back, neck, and thorax regions and in high numbers in the abdomen and limb regions (Fig. 6). The abdomen had the highest melanocyte count, with significant differences when compared to the neck, back, and thorax. Moreover, the neck region had the lowest count (Fig. 6). No significant differences were observed among limb, back, and thorax, suggesting similar melanocyte counts in these regions (Fig. 6).

## Discussion

The donkey has gained great attention due to the most cost-effective sources of transport and its ability to tolerate drought conditions. Some economic problems were linked to this species, such as decreasing their number compared to the rising demand for donkey hide in the Chinese market. The epidermal layer, with its cornified epithelium outer covering, provided skin strength and resistance against scraped areas, while the delineated squamous epithelium inner coating provided protection.

The current findings reveal that the epidermal part at the top of the apices of the dermal papillae gave rise to various villous protrusions in the dermis, giving rise to a serrated appearance in the abdomen, back, and neck regions. Our findings reveal the histomorphological characteristics of five different regions of *Equus asinus* skin using both appropriate staining techniques for light microscopy and precise identification by TEM microscopy, yielding a great deal of new data, including collagen content in donkey’s skin and skin thickness in various regions. Initially, the donkey’s skin is composed of two layers, the epidermis and dermis. The skin epidermis is composed of four layers, comprising the stratum corneum, stratum basale, stratum spinosum, and stratum granulosum, such as those of a horse [16, 24], Brandt’s hedgehog [8], Bakerwali goat [25], and ostrich [26], whereas the epidermis of the pigs has a three-layered epidermis: stratum germinativum, stratum granulosum, and stratum corneum, with the absence of stratum lucidum [27, 28], and also the deer has only three layers [29]. However, according to Hanafy, et al. [30], the domestic pig’s epidermis comprises four layers: stratum corneum, stratum granulosum, stratum spinosum, and stratum basale.

Melanocytes, a type of cell in the basal layer of the epidermis, are found in various parts of the body, including hair follicles, sebaceous and sweat gland ducts, and the superficial dermis, produce the skin and hair coloring pigment melanin [31]. In this study, melanocytes are observed in a low number within the stratum basale of the back, neck, and thorax regions and a high number in the abdomen and limb regions, and this is inconsistent with the finding of Sathapathy, et al. [32] that the zebra’s limb region has the highest amount of melanin deposition, followed by the abdomen and thighs. The current findings reveal that the basal cells in the epidermal part atop the apices of the dermal papillae give rise to various villous cytoplasmic protrusions in the dermis, giving rise to a serrated appearance in the abdomen, back, and neck regions. These dermal papillae name the papillomatous epidermis are also described in numerous animal species such as donkey, buffalo, pig, camels, sheep, and dog, but really observed in cow and goat [33]. The current findings concludes that the dermo-epidermal interface has a wavy form with well-defined columns of dermis protruding in-between dermal papillae, while Tong, et al. [34] described them as rete ridges.

Further, the dermis has been differentiated into an outer, thinner papillary layer and an inner, thicker reticular layer, which constitute the superficial dermis and deep dermis, respectively. These findings are consistent with those recorded in horses [16, 35, 36], donkeys, buffalo, cows, pigs, camels, sheep, goats, and dogs [33], and cattle [37]. It is also noted that the skin has a dermis, which is composed of fibers that give the skin its elasticity and support for surrounding tissues [16, 38]. In particular, our study recorded three fibrous layers of the dermis, comprising the papillary layer, reticular layer, and cordovan layer. The current findings are in harmony with Jorgensen, et al. [16] who mentioned that below the reticular dermis is a cordovan layer, but collagen becomes thicker, forming the third accessory layer. The current findings indicate the presence of a parallel, deeper, denser collagen layer (the third dermal layer) in the limb named the accessory Cordovan layer, and this is similar to the observations stated by Wakuri, et al. [35], Ahmed, et al. [39] in the horse. This cordovan layer gives the (horse mirror) appearance of skin, as reported by Talukdar, et al. [40].

In this study, the hair follicles have an oval shape, but in contrast, they have a rectangular shape in Indian bison [41], but generally they are observed in an oval shape in cows and goats [33, 42]. Primary follicles in buffalo, cattle, horses, and goats are arranged in linear rows, randomly distributed in pigs, and compound-type in capsular form in dogs [43]. The current findings reveal that the sweat glands are found in the central part of the reticular layer or deeper. These sweat glands are also observed in different animal species: donkeys, buffalo, pigs, camels, sheep, goats, and dogs, but are really observed in cows and goats [33]. On the contrary, the sweat glands are not detected in the zebras’ skin, as reported by Shambhulingappa, et al. [41]. Additionally, Yang, et al. [44] found that the sweat glands in yaks’ skin are poorly developed. The number of sweat glands varies greatly between regions, indicating a role in thermoregulatory evaporative cooling, particularly in hot climates [45], besides participating in transdermal drug delivery [46].

The current findings found that the arrector pili muscle is a small smooth muscle bundle near the beginning of the epidermis, and then at the sebaceous gland, it is divided into as many branches similar to those reported in the horse by Talukdar, et al. [40] and in different animal species such as donkeys, buffalo, pigs, camels, sheep, goats, and dogs, but are really observed in cows and goats [33]. In the dermal papillary layer, the arrector pili muscles are typically located around the hair follicle and are always linked to the sebaceous gland [47]. In cold weather, the contraction of these muscle fibers traps air inside the coat through the elevated hair, insulating the body [32]. They also stimulate other secretion products in the secretion channel. Our findings found simple, branched sebaceous glands in the dermis’ reticular layer, with a multi-bubble cytoplasm and central nuclei, and sebaceous glands with sacculations accompanying oval-shaped hair follicles in various anatomical regions, similar to that described in some animal species such as donkeys, buffalo, cows, pigs, camels, sheep, goats, and dogs [33]. Meanwhile, the nose of Brandt’s hedgehog has more sebaceous glands than any other area, but none are present in the cloak area [8].

In the present study, we initially measured the epidermal and dermal thickness in various areas of the skin of the donkey. The thinnest skin is observed at the limb, while the thickest skin is at the back, which corresponds to equine skin, as evidenced by [35], Volkering [48], and as previously mentioned in Yak by [44], Zhang [49]. This is a result of the back being the body part most exposed to storms, snowfall, and rainfall. In the trunk, the thickness of the donkey skin decreased from the dorsal to the ventral. The majority of domesticated large animals exhibited this pattern of variation in skin thickness, as stated by Danny [50]. On the other hand, the epidermis and dermis of the limb in zebras are thicker when compared with the abdomen [32].

The current findings reveal that the dermal-epidermal junction of the back skin of the donkey is significantly longer than that of the other skin regions examined. This is significant because the dermo-epidermal interface facilitates a strong connection between the epidermis and the dermis and ensures a foundation for re-epithelialization during wound healing, helping to maintain barrier function either to or from the epidermis [51].

Collagen constitutes a significant portion of the epidermis, and variations in its thickness with age have been linked to the amount of collagen in the skin [33, 52]. According to our current investigation, the collagen fibers’ crisscrossing in the third layer of the dermis may have a remarkable supporting role in the elastic rebound mechanism. This redirects the stores and lowers the amount of energy used for movement [53]. The variation in the thickness, extent, and pigmentation patterns of the skin and the distribution of skin appendages in various body areas led to the uniqueness of this species of animal.

Another unique aspect of the current study is the inclusion of ultra-structural observation of the skin of *Equus asinus*. Generally, awareness of the average thickness of a donkey’s skin and collagen contents may be helpful in collecting skin grafts for leather production and the cosmetics industry, respectively. Furthermore, these results are helpful for better understanding and appreciating the adaptability features of donkey skin.

## Conclusion

From the current investigation, it can be concluded that the thinnest skin is the skin at the limb, while the thickest one is at the back, in addition to the presence of a third accessory layer in the skin of the donkey’s limb called the accessory Cordovan layer. Moreover, it was noted that the skin of the thoracic region contains a high percentage of collagen, but it is not significant. The study explores the ultra-structural composition of donkey skin, offering insights into its resilience to environmental conditions and potential biomedicine applications, benefiting various industries and research fields related to *Equus asinus*.

## Electronic supplementary material

Below is the link to the electronic supplementary material.

Supplementary Material 1

Supplementary Material 2

Supplementary Material 3

Supplementary Material 4

Supplementary Material 5

Supplementary Material 6

## Data Availability

The datasets used and/or analyzed during the current study are available from the corresponding author on reasonable request.
