# Supplementary figures and images for: Elucidation of collagen content in different anatomical regions of the dermis of donkeys (Equus asinus): histomorphometric and ultrastructural study
Source: BMC Vet Res. 2025 May 2;21:310. doi: 10.1186/s12917-025-04712-0 (PMC12046934; doi:10.1186/s12917-025-04712-0)

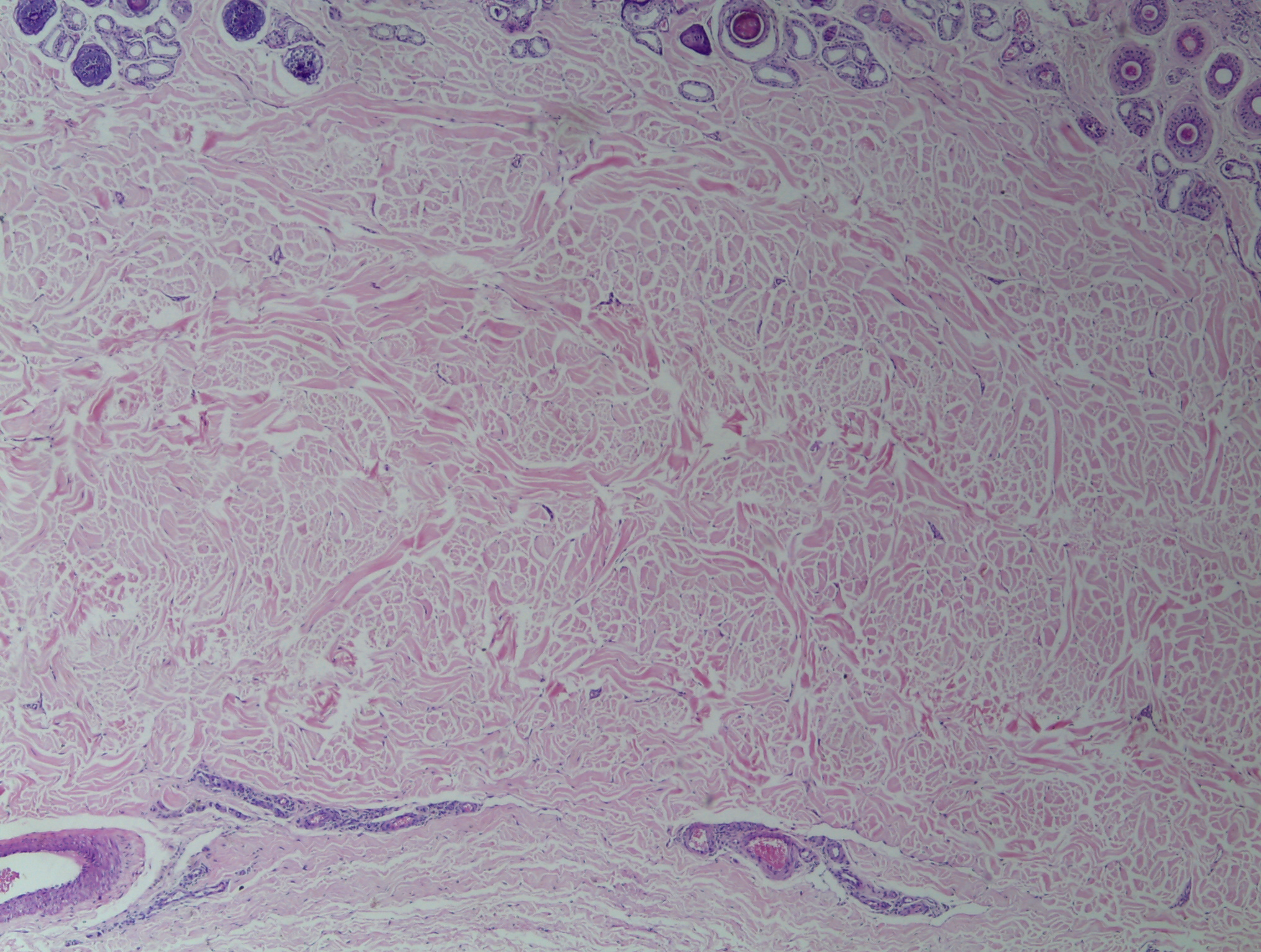

Supplement: Supplementary file 1 — Supplementary Material 1 [file 12917_2025_4712_MOESM1_ESM.tif]

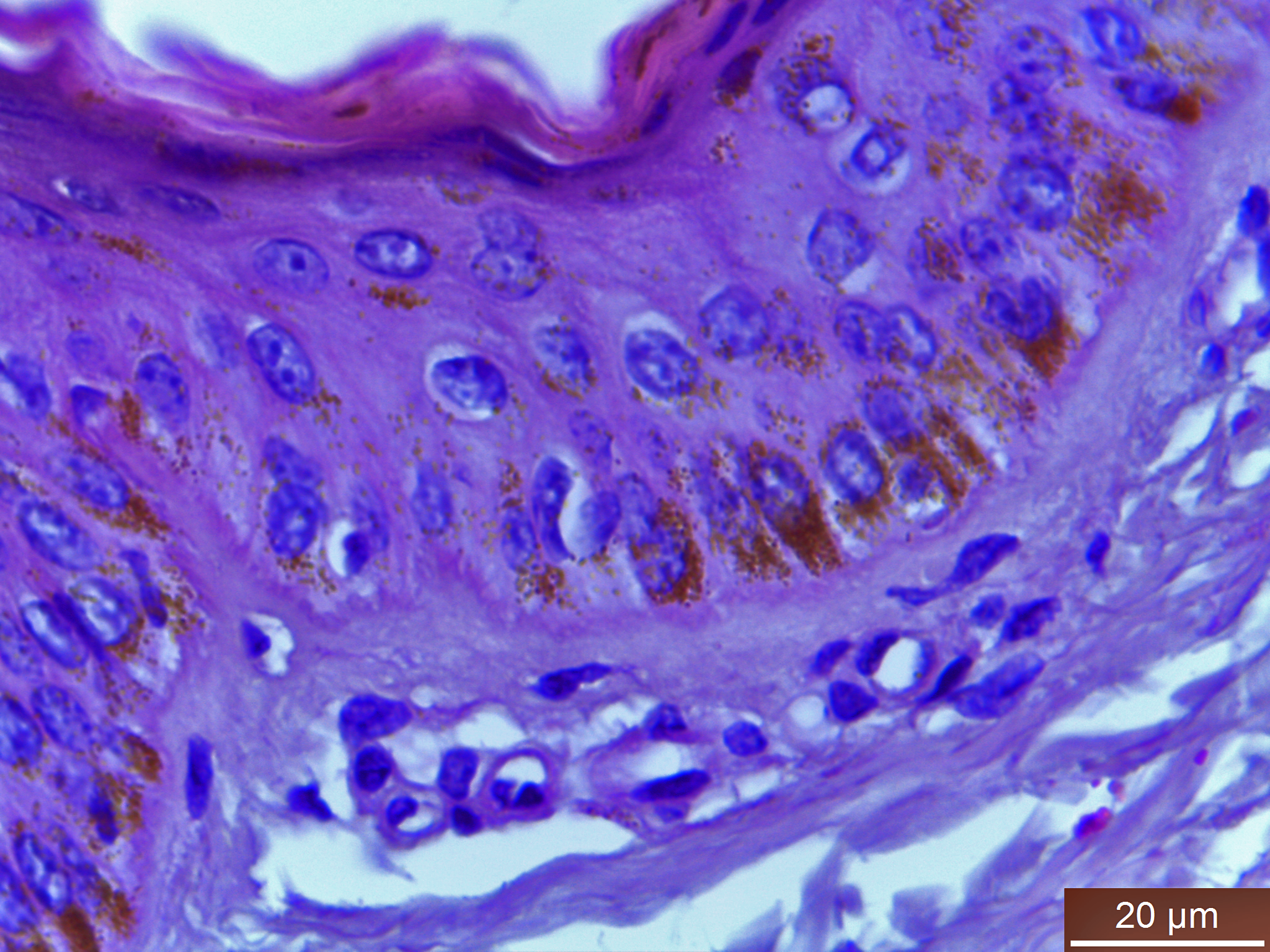

Supplement: Supplementary file 2 — Supplementary Material 2 [file 12917_2025_4712_MOESM2_ESM.tif]

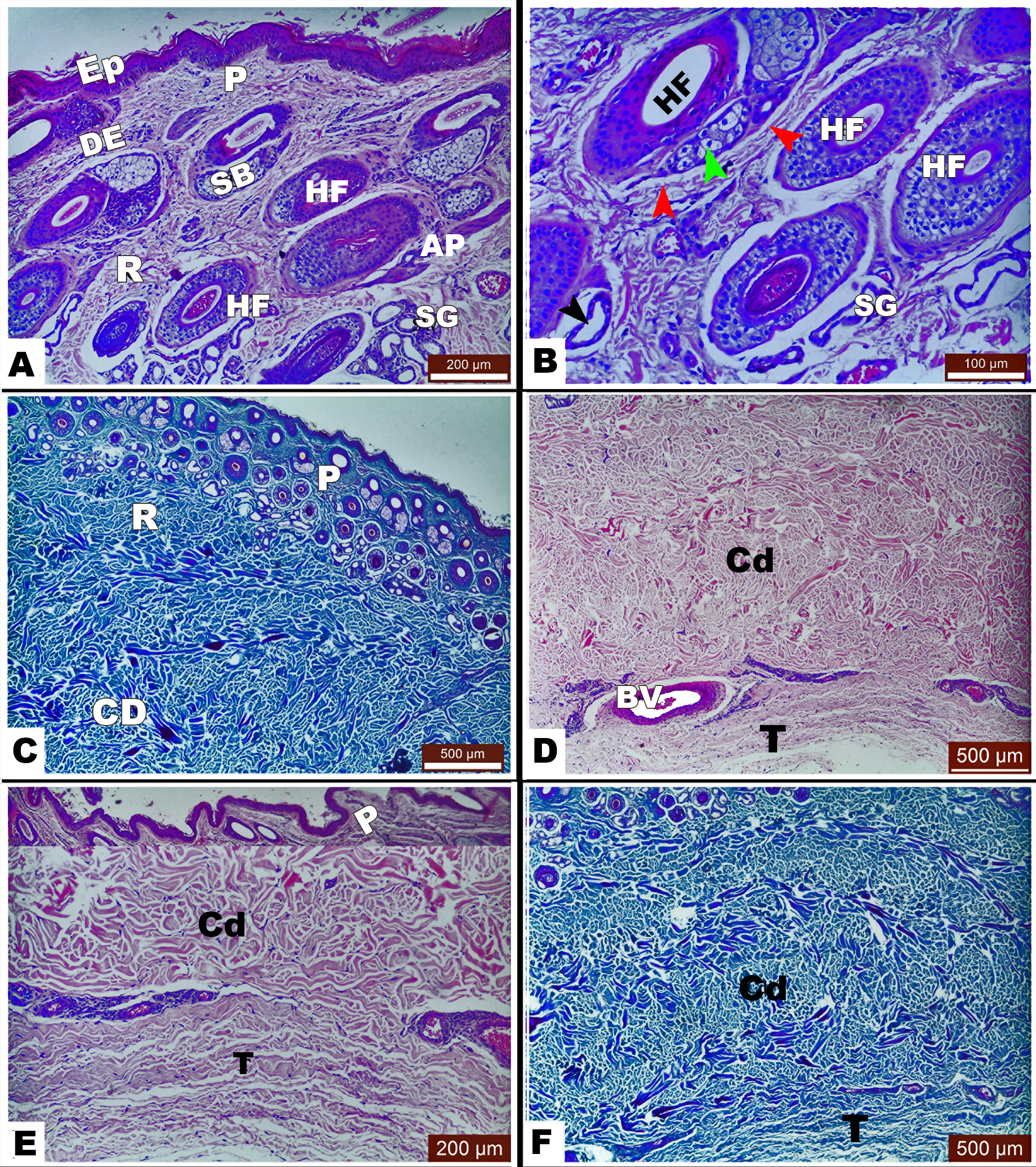

Supplement: Supplementary file 3 — Supplementary Material 3 [file 12917_2025_4712_MOESM3_ESM.tif]

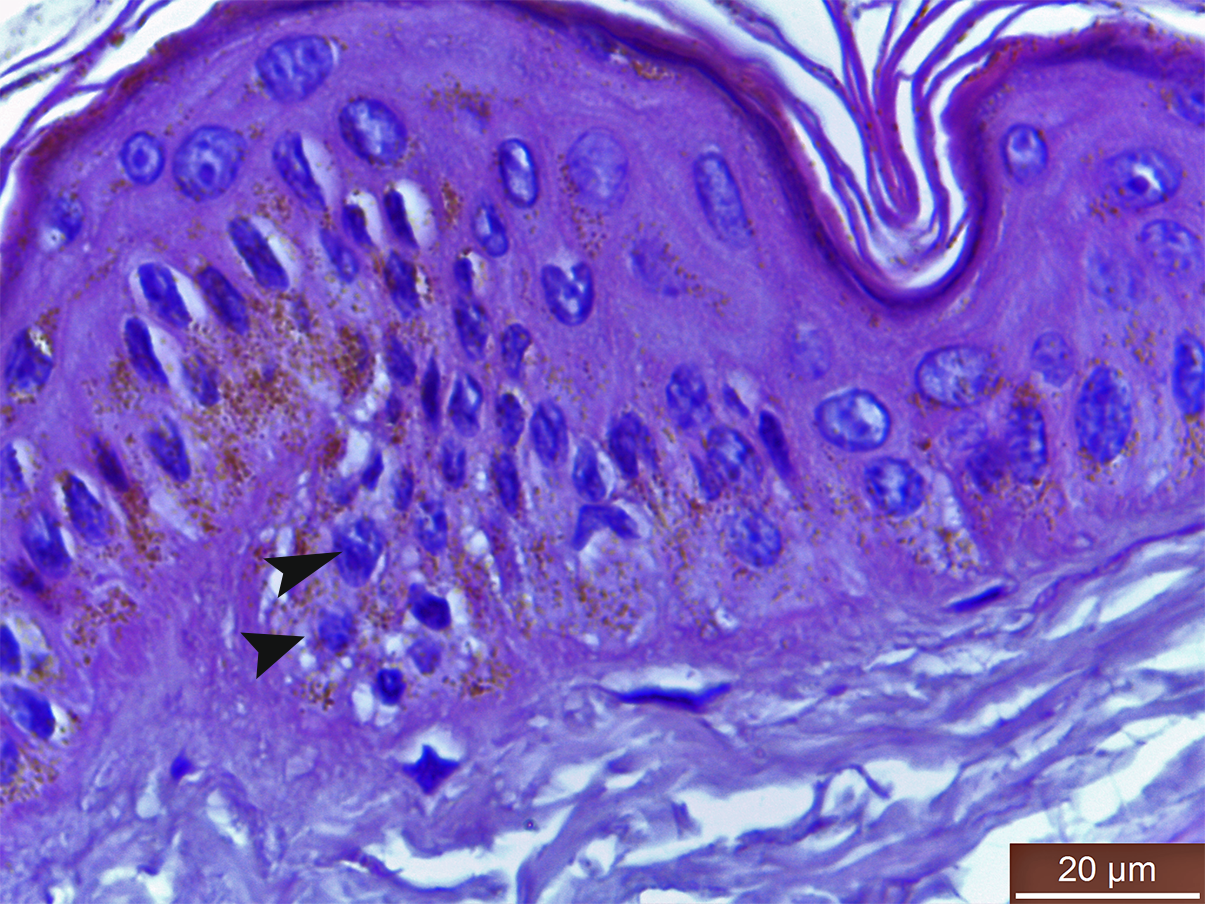

Supplement: Supplementary file 4 — Supplementary Material 4 [file 12917_2025_4712_MOESM4_ESM.tif]

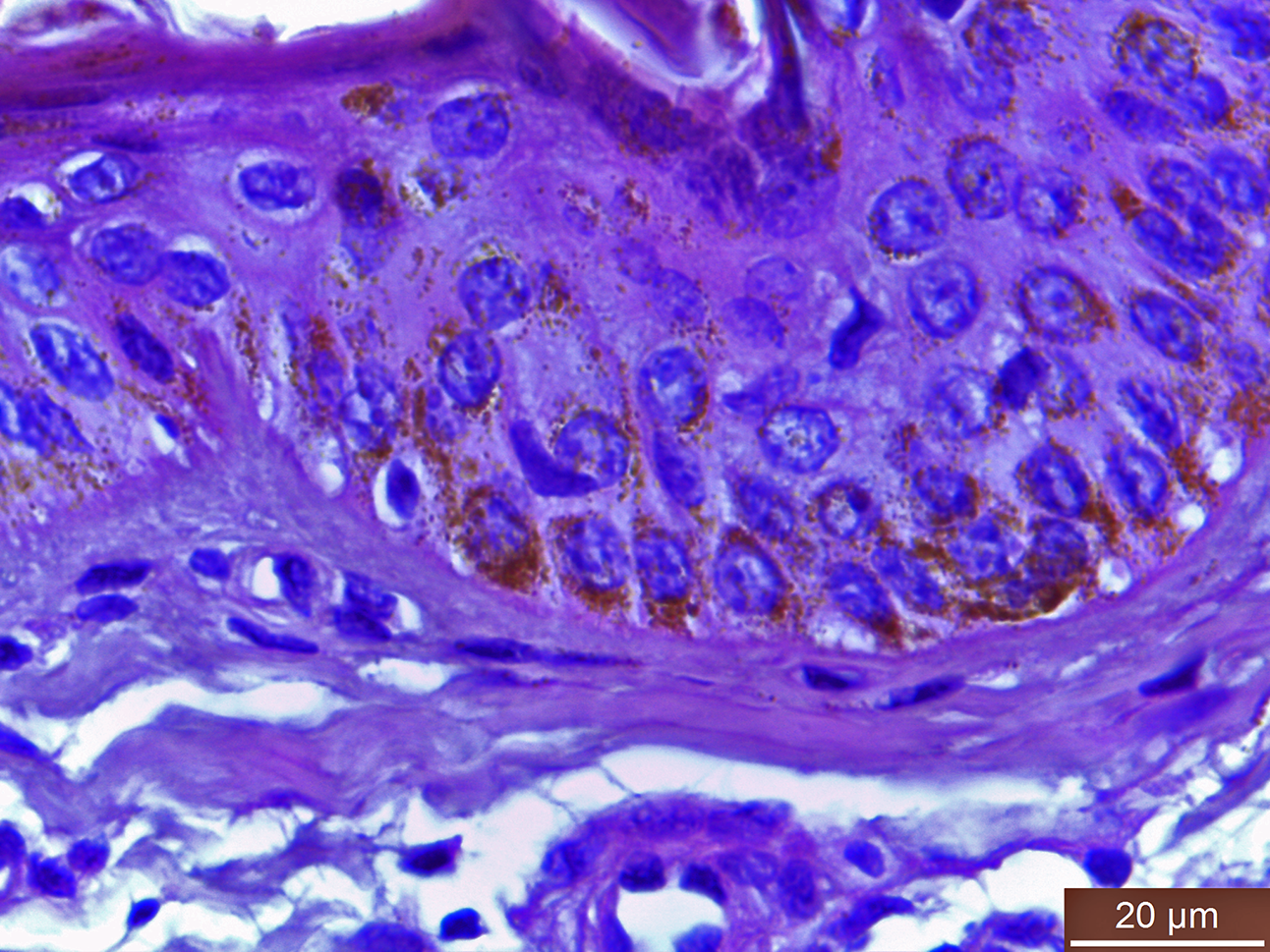

Supplement: Supplementary file 5 — Supplementary Material 5 [file 12917_2025_4712_MOESM5_ESM.tif]

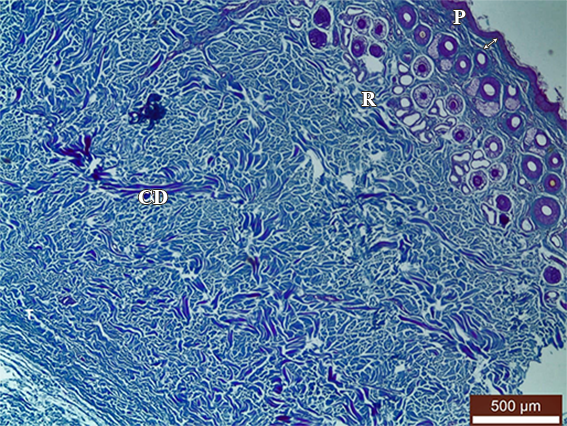

Supplement: Supplementary file 6 — Supplementary Material 6 [file 12917_2025_4712_MOESM6_ESM.tif]
